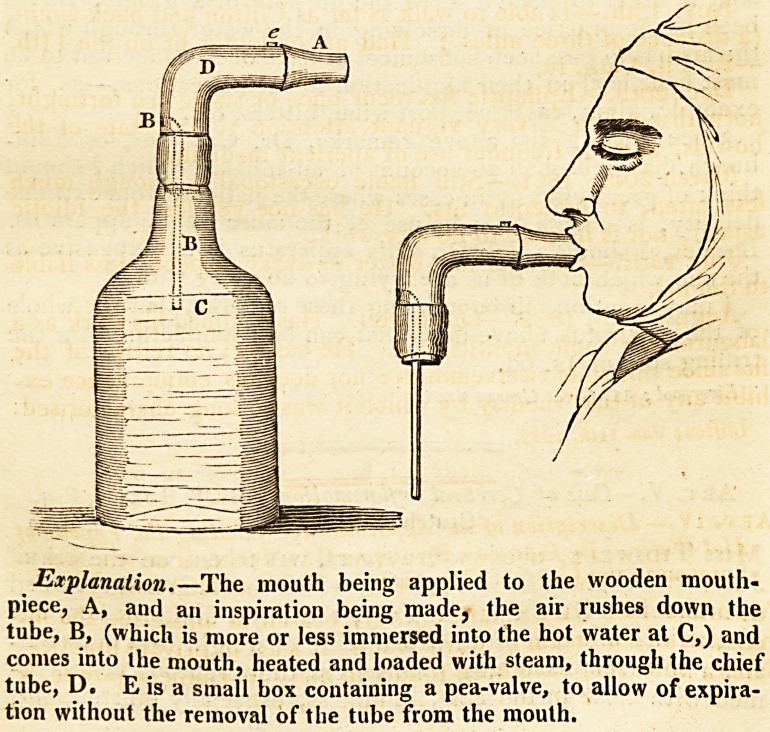# Description of an Apparatus for Inhaling the Vapour of Hot Water

**Published:** 1823-09

**Authors:** George Douglas Cameron

**Affiliations:** Surgeon to the Liverpool Dispensary.


ART. IV.—*Description of an Apparatus for inhaling the Vapour of Hot Water.* By GEORGE DOUGLAS CAMERON, M.D. M.R.M.S.E. Surgeon to the Liverpool Dispensary. [With an Engraving.]

MEDICAL men have almost entirely ceased to prescribe the inhalation of the steam of warm water, at least in private practice. None, however, have been found to deny its eminent power, if properly applied, both as a diaphoretic and in alleviating some of the most disagreeable symptoms of cynanche and catarrh. In thinking of the circumstances which could give rise to the discontinuance of this excellent practice, I at once fixed upon the clumsiness and expensiveness of the common apparatus: for, in prosecuting some trials with respect to croup, I soon found that no diaphoretic effect could possibly be produced by this most inefficient of inventions.

In considering how the disadvantages of this apparatus were to be done away with, and to render this excellent practice more general, the following circumstances appeared at once as the most necessary to be obviated: that, in the common apparatus, it is only the steam arising spontaneously which is inhaled by the patient; and that this spontaneous vapour, being in such small quantity in water below 212° of Fahrenheit, passes with many times its bulk of cold air along a flexible tube of at least a foot in length. In revolving these objections, and having the opportunity of seeing a very ingenious, but imperfect, invention, by my late friend Mr. Hercy, while domestic physicians in the Edinburgh Infirmary, I was lead to the contrivance delineated in the annexed engraving.

*Explanation.*—The mouth being applied to the wooden mouthpiece, A, and an inspiration being made, the air rushes down the tube, B, (which is more or less immersed into the hot water at C,) and comes into the mouth, heated and loaded with steam, through the chief tube, D. E is a small box containing a pea-valve, to allow of expiration without the removal of the tube from the mouth.

With regard to the facilities and efficacy of this plan of administering the vapour of warm water, I have had most ample proofs: while medical superintendent of the Queensberry-house Hospital in Edinburgh, I tried the method most extensively. In fever cases of the worst nature, when the mouth and fauces were dry and horny, this afforded most marked relief. I caused the bottle to be nearly filled with boiling water, so that the tube, B, should be about an inch and a half in the fluid, and rolled in a piece of flannel; the tube, A, being inserted into the patient's mouth, and the bottle laid with its neck resting against the pillow: thus allowing the patient to breathe the steam without any material exertion, or even any alteration of position.

I have not alone my own experience of the sufficiency of this invention: my friend Mr. Hennen, the distinguished author of "Military Surgery," and inspector of hospitals, was so kind as to try these inhalers in Edinburgh Castle; and was so much pleased with the facility of their use, that he sent one, with recommendations, to Sir James M'Grigor, the director-general.

Of late I have further extended the use of this simple machine to the cure or alleviation of mercurial sore throat and mouth; and, in order to make it serve the purpose of a gargle, I have been in the practice of dissolving in the water through which the air has to pass such substances as have been conceived to be most beneficial in their application to such affections,—as, for example, alum, cayenne, port wine, bitters, &c.

Since writing the above remarks, Dr. Gardiner, of Edinburgh, has published an account of an inhaler, which I should think of great efficacy in cases where the patient, from extreme debility, is unable to make use of the more simple apparatus. Besides, I should think Dr. G.'s apparatus is as expensive as the one which both of us are trying to do away with.

I may mention, in concluding these remarks, that the whole of the apparatus above described can be manufactured for the trifling sum of 1s. 6d.

***Liverpool; 41, Great George-street.***

## Figures and Tables

**Figure f1:**